# Thermoelectric Properties of Ca_1−*x*_Gd_*x*_MnO_3−**δ**_ (0.00, 0.02, and 0.05) Systems

**DOI:** 10.1100/2012/149670

**Published:** 2012-09-10

**Authors:** Ankam Bhaskar, Chia-Jyi Liu, J. J. Yuan

**Affiliations:** Department of Physics, National Changhua University of Education, Changhua 500, Taiwan

## Abstract

Polycrystalline samples of Ca_1−*x*_Gd_*x*_MnO_3−*δ*_ (*x* = 0.00, 0.02, and 0.05) have been studied by X-ray diffraction (XRD), electrical resistivity (**ρ**), thermoelectric power (S), and thermal conductivity (**κ**). All the samples were single phase with an orthorhombic structure. The Seebeck coefficient of all the samples was negative, indicating that the predominant carriers are electrons over the entire temperature range. The iodometric titration measurements indicate that the electrical resistivity of Ca_1−*x*_Gd_*x*_MnO_3−*δ*_ correlated well with the average valence of Mn^*v*+^ and oxygen deficiency. Among the doped samples, Ca_0.98_Gd_0.02_MnO_3−*δ*_ had the highest dimensionless figure of merit 0.018 at 300 K, representing an improvement of about 125% with respect to the undoped GaMnO_3−*δ*_ sample at the same temperature.

## 1. Introduction

Thermoelectric generators can convert waste heat into electric energy without using moving parts and without producing carbon dioxide gas, toxic substances, or other emissions. It is expected that thermoelectric power generation can provide a new energy source from the conversion of waste heat emitted by automobiles, factories, and other similar sources. For this purpose, oxide materials are potential candidates for a wide range of high-temperature applications due to their high chemical stability and the absence of harmful elements in their compositions. Since the discovery of large thermoelectricity in Na_*x*_CoO_2_ [1], enthusiastic efforts have been devoted to explore new oxides exhibiting high thermoelectric performances, and some layered cobaltites such as [Ca_2_CoO_3_][CoO_2_]_1.62_ and [Bi_0.87_SrO_2_]_2_[CoO_2_]_1.82_ are found to exhibit interesting thermoelectric properties [2–4]. These p-type materials present large thermopower (>100 *μ*V/K at 300 K), a relatively low electrical resistivity (~1–10 mΩ–cm at 300 K), and are expected to be incorporated in thermoelectric modules. More recently, theoretical predictions lead interest to focus on low-dimensional materials with a ZT of 2.4 in Nb-doped SrTiO_3_ thin films due to the giant Seeback coefficient in the superlattices [5]. On the other hand, the intensive investigations on n-type oxides are still going on because their lower performances compared to p-type materials. Among these n-type materials, the perovskite CaMnO_3_-based compound has been intensively studied due to its relatively low electrical resistivity and high Seebeck coefficient [6–10]. The decrease in the electrical resistivity by the means of cationic substitutions in the “A” site, like in R_1−*x*_A_*x*_MnO_3_ [8] (R: rare earth cation, A: divalent cation such as Ca, Sr, Ba, and Pb), La_1−*x*_Sr_*x*_MnO_3_ [9], Ca_1−*x*_Dy_*x*_MnO_2.89_ [11], and Ca_1−*x*_La_*x*_MnO_3_ [12]. Tang et al. [13] reported that Gd substitution of Ca_3−*x*_Gd_*x*_Co_4_O_9+*δ*_ improved the figure of merit, which was about one order of magnitude larger than that of Ca_3_Co_4_O_9+*δ*._

In these studies, we report thermoelectric properties of Ca_1−*x*_Gd_*x*_MnO_3−*δ*_ (0.00, 0.02, and 0.05) systems.

## 2. Experimental Details

Polycrystalline samples of Ca_1−*x*_Gd_*x*_MnO_3−*δ*_ (0.00, 0.02, and 0.05) were synthesized by the solid-state reaction from CaCO_3_, Mn_2_O_3_, and Gd_2_O_3_ powders. The powders were heated at 1173 K for 10 h and at 1473 K for 20 h in air with intermediate grinding. The resulting powders were then pressed into parallelepiped and sintered in air at 1473 K for 20 h. The phase purity of resulting powders was examined by a Shimadzu XRD-6000 powder X-ray diffractometer equipped with Fe *Kα* radiation. Electrical resistance measurements were carried out using standard four-probe techniques. Thermopower measurements were performed between 300 and 700 K using a steady-state technique with a temperature gradient of 0.5–2 K across the sample. A type E differential thermocouple was used to measure the temperature difference between the hot and cold ends of the sample, which was measured using Keithley 2000 multimeter [14]. The temperature difference was typically between 0.5 and 1 K. The thermopower of the sample was obtained by subtracting the thermopower of Cu Seebeck probes. Thermal conductivity measurement was carried out using transient plane source techniques with very small temperature perturbations of the sample material by the hot disk thermal constants analyzer. The transient plane source technique makes use of a thin sensor element in the shape of a double spiral. The hot disk sensor acts as both a heat source for generating temperature gradient in the sample and a resistance thermometer for recording the time-dependent temperature increase [15]. The encapsulated sensor was sandwiched between two pieces of samples. During a preset time, 200 resistance recordings were taken and from them a relation between temperature and time was established. The oxygen contents and valence state of manganese were determined using iodometric titration [16].

## 3. Results and Discussion

**Figure 1:**
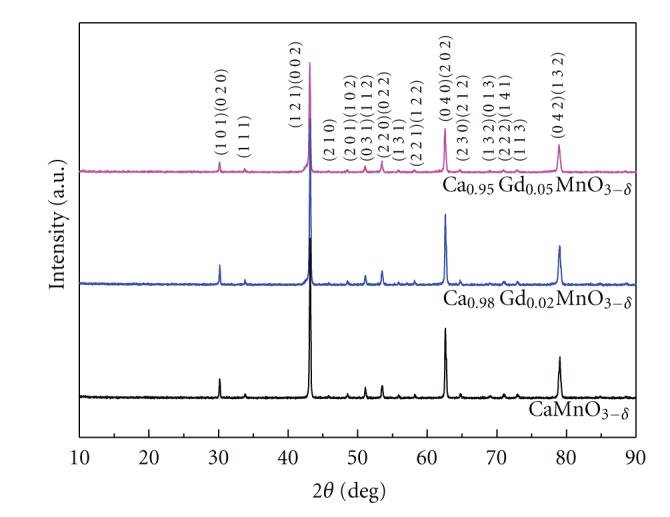
XRD patterns of the Ca_1−*x*_Gd_*x*_MnO_3−*δ*_ (0.00, 0.02, and 0.05).

XRD analysis indicates that all the samples of Ca_1−*x*_Gd_*x*_MnO_3−*δ*_ (0.00, 0.02, and 0.05) are a single phase with orthorhombic symmetry of *Pnma*. The typical XRD patterns are shown in Figure 1. The diffraction peaks are matched with earlier reports of CaMnO_3_ [17], and no secondary phase is observed. The similarity between the crystal structures of undoped and doped samples suggests that the doped ions do not change the crystalline structure. Lattice parameters are calculated and tabulated in Table 1. As seen in Table 1, the lattice parameters do not show monotonic trend, which may be due to the oxygen deficiency and small amount of dopants. Trukhanov et al. [18] reported that lattice parameters changed due to the oxygen deficiency in La_0.50_Ca_0.50_MnO_3−*δ*_ (0 ≤ *δ* ≥ 0.50). Wiebe et al. [19] also reported that lattice parameters changed due to the oxygen deficiency in CaMnO_3−*δ*_ (*δ* = 0.06,0.11).

Table 2 summarizes the characterization and properties of the samples at room temperature. The undoped sample shows the highest resistivity among the samples, while the resistivity of the doped samples is significantly lower than for the undoped sample due to the substitution of trivalent Gd^3+^ for divalent Ca^2+^, which decreases the concentration of holes. The value of *ρ* for doped samples is in the range of 0.041 Ω–cm to 0.019 Ω–cm, which increases with increasing dopant content. For doped samples, the average valence of Mn^*v*+^ decreases and oxygen deficiency increases as compared to undoped sample. Doping of the Ca site with Gd causes a strong decrease of the *ρ* due to the creation of charge carrier content of Mn^3+^ in the Mn^4+^ matrix. The concentration of carriers in these samples can be correlated with the oxidation state of Mn. Creation of Mn^3+^ comes from two sources in the title system, that is, doping of Gd^3+^ and oxygen deficiency. Oxygen deficiency in CaMnO_3−*δ*_ creates two Mn^3+^ five-coordinate sites for each O vacancy according to the X-ray absorption near-edge spectra results [20]. In order to compensate the oxygen deficiency and maintain the electrical neutrality, the creation of Mn^3+^could be present in these samples. There are also earlier reports, an oxygen deficiency for other electron-doped calcium manganites [21, 22]. The negative thermopower confirms that the dominant charge carriers are electrons for all the samples. The undoped CaMnO_3−*δ*_ has a very large absolute S, being about −319 *μ*V/K at 300 K. The room temperature absolute value of S for the undoped sample is lower than the value of −600 *μ*V/K reported by Flahaut et al. [23], which may be due to the oxygen deficiency. The doped samples show relatively small absolute S due to the increase of carrier concentration (Table 2). The doped sample induces a clear decrease of the absolute *S* value due to the increase of the concentration of Mn^3+^.

**Figure 2:**
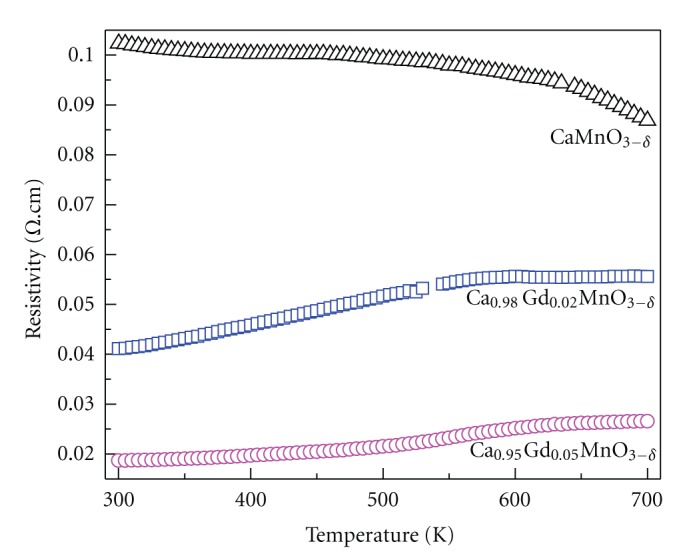
The temperature dependence of the electrical resistivity of the Ca_1−*x*_Gd_*x*_MnO_3−*δ*_ (0.00, 0.02, and 0.05).

The temperature dependence of resistivity of Ca_1−*x*_Gd_*x*_MnO_3−*δ*_ (0.00, 0.02, and 0.05) is shown in Figure 2. The undoped sample exhibit nonmetallic behavior in the entire temperature range, that is, the resistivity decreases with increasing temperature (d*ρ/*d*T < *0). Similar tendency was also reported for undoped sample (CaMnO_3−*δ*_) [23]. The doped samples show that the resistivity increases with increasing temperature in the whole temperature range, indicating the metallic behavior (d*ρ/*d*T* > 0). This is a similar behavior to that of the electron-doped manganites above the metal-insulation transition temperature [24, 25].

**Figure 3:**
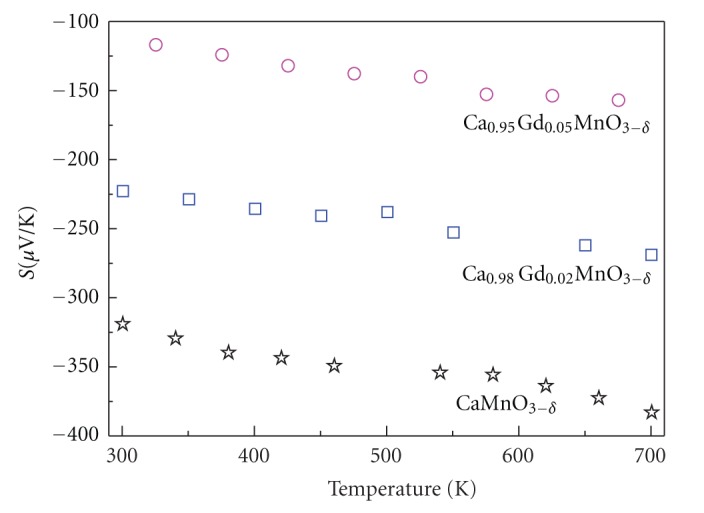
The temperature dependence of the Seebeck coefficient S of the Ca_1−*x*_Gd_*x*_MnO_3−*δ*_ (0.00, 0.02, and 0.05).

Figure 3 shows the Seeback coefficient (S) as a function of temperature (300–700 K) for Ca_1−*x*_Gd_*x*_MnO_3−*δ*_ (0.00, 0.02, and 0.05). All samples exhibit negative values of the thermopower, which indicates that the electrons are the predominant charge carriers (n-type conduction). The absolute thermopower increases with increasing temperature and exhibit metallic behavior for undoped sample, which is contrast to Ohtaki's sample [26]. Ohtaki et al. [26] reported that absolute value of thermopower decreases with increasing temperature, typical characteristic of nonmetal-like temperature dependence. This difference should be attributed to the contribution of the oxygen deficiency [27].

For materials with more than one type of charge carrier, the diffusion thermopower can be expressed as

(1) $$\begin{array}{c} S=\sum_{i}\left(\frac{\sigma_{i}}{\sigma }\right)S_{i}, \end{array}$$

where *σ*_*i*_ and *S*_*i*_ are the partial electrical conductivity and partial thermopower associated with the *i*th group of carriers, respectively. We can rewrite thermopower of CaMnO_3−*δ*_ and as

(2) $$\begin{array}{c} S=\frac{\sigma_{\text{in}}}{\sigma_{\text{in}}+\sigma_{\text{ex},\text{defect}}}S_{\text{in}}+\frac{\sigma_{\text{ex},\text{defect}}}{\sigma_{\text{in}}+\sigma_{\text{ex},\text{defect}}}S_{\text{ex},\text{defect}}, \end{array}$$

where *σ*_in_ and *S*_in_ are the contribution from intrinsic carriers; *σ*_ex,defect_ and S_ex,defect_ are the contribution from extrinsic carriers due to oxygen defects. Since the increase of electrical conductivity (~*e* ^−*E*_*a*_/*k*_*B*_*T*^) is faster than the decrease of *S *(~*E*_*a*_/*k*_*B*_ *T*) for semiconductors, one could expect that the second term in (2) would increase and therefore the absolute value of thermopower for CaMnO_3−*δ*_ would increase, which should be responsible for the simultaneous increase of the electrical conductivity and absolute value of thermopower with increasing temperature.

**Figure 4:**
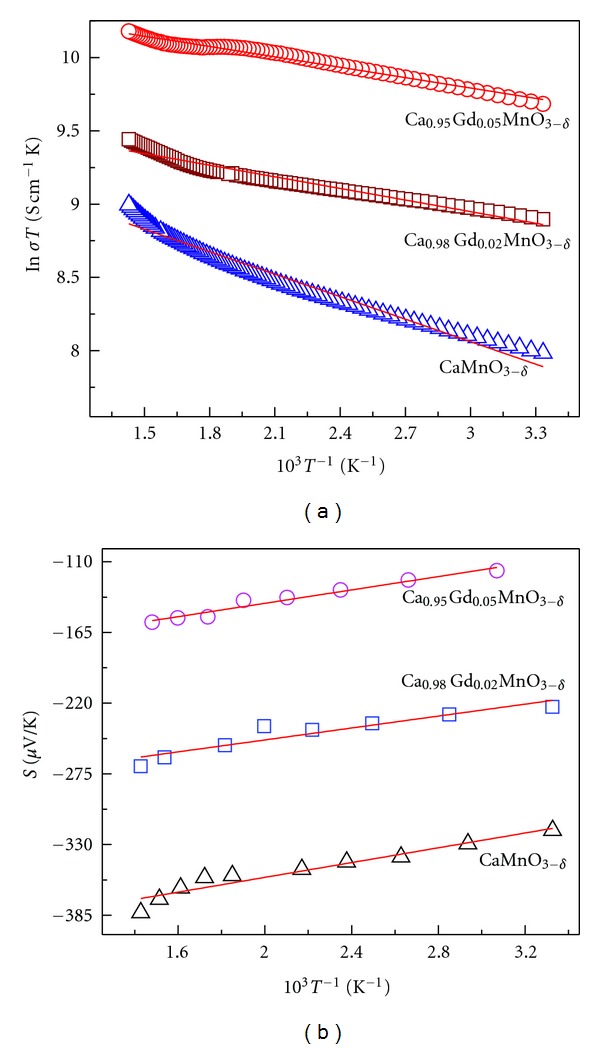
Plots of (a) In (**σ*T*) versus 1000/T and (b) S versus 1000/T of the Ca_1−*x*_Gd_*x*_MnO_3−*δ*_ (0.00, 0.02, and 0.05).

The experimental activation energies derived from electrical resistivity and thermopower measurements are distinguishable for the title system, and the adiabatic small polaron conduction model [28] has often been invoked to account for the transport mechanism in both electrical resistivity and thermopower [24, 26, 29].  Figure 4 shows similar fitting for Ca_1−*x*_Gd_*x*_MnO_3−*δ*_ (0.00, 0.02 and 0.05) in the present study using the following forms *ρ* = *ρ*_0_ *T *exp(*E*_*a*_/*k*_*B*_ *T*) and *S *= (*k*_*B*_ */e*)(*W*_*H*_/*k*_*B*_ *T*+ *B*), where *E*_*a*_ is one-half of the energy gap between the polaronic bands, *W*_*H*_ is one-half of the polaron binding energy, *B* is associated with the spin and the mixing entropy, and *e* is the electron charge with minus sign. One would expect a decrease of absolute value of thermopower with increasing temperature and obtain a negative *W*_*H*_ when fitting thermopower data for the polaronic transport. 

The thermal conductivity is measured at room temperature, and values are presented in Table 2. Total thermal conductivity (*κ*_total_) can be expressed as

(3) $$\begin{array}{c} \kappa_{\text{total}}=\kappa_{\text{el}}+\kappa_{\text{ph}}, \end{array}$$

where *κ*_el_ and *κ*_ph_ represent the electronic and lattice thermal conductivity, respectively. *κ*_el_ can be calculated by using the Wiedemann-Franz-Lorenz relationship

(4) $$\begin{array}{c} \kappa_{\text{el}}=L\sigma T, \end{array}$$

where *L* = *π* ^2^ *k* ^2^/3*e* ^2^ = 2.45 × 10^−8^ W Ω K^−2^ is the Lorenz number and *T* is the absolute temperature. *κ*_ph_ is obtained by subtracting *κ*_el_ from *κ*_total_. It can be clearly seen from Table 2 that the total thermal conductivity for all the doped samples is less than that of CaMnO_3−*δ*_. For materials with *ρ* > 1 Ω–cm, *κ*_el_ is negligible. But in our case, the resistivity is lower than 1 Ω–cm, a fact which leads us to determine the *κ*_el_ by using the Wiedemann-Franz law. The calculated value of *κ*_el_, for CaMnO_3−*δ*_ is 0.019 W m^−1 ^K^−1^ and Ca_0.95_Gd_0.05_MnO_3−*δ*_ is 0.027 at 700 K, respectively. For all the samples, the lattice contribution is more important than the electronic one. Due to the small *κ*_el_, *κ*_total_ is mainly attributed to the lattice contribution. The *κ*_total_ of Ca_0.95_Gd_0.05_MnO_3−*δ*_ is 1.26 at 300 K, being an indication of these doping effects. It should be emphasized that in contrast to the CaMnO_3−*δ*_ and the Ca_0.95_Gd_0.05_MnO_3−*δ*_, samples show much lower *κ*_total_. For all doped samples, the lattice contribution is more important than the electronic one. Due to the small *κ*_el_, *κ*_total_ is mainly attributed to the lattice contribution. Both of the *κ*_el_, *κ*_ph_ decrease with increasing dopant content (Table 2). The radius and mass of Gd^3+^ and Ca^2+^ ions are different, and substitution of Gd^3+^ and Ca^2+^ can affect the value of *κ*_ph_. The effect of Gd^3+^ doping on the lattice vibration arises from two main factors. One is the crystallographic distortion and the other is the mass difference between Gd^3+^ and Ca^2+^ [24].

The power factor (*S* ^2^ *σ*) is calculated and presented in Table 2. The highest value of *S* ^2^ *σ* (1.21 *μ*W cm^−1 ^K^−2^ at 300 K) is obtained for Ca_0.98_Gd_0.02_MnO_3−*δ*_. The figure of merit (*ZT* = *S* ^2^ *σT*/*κ*) is calculated for all the samples. The calculated values are presented in Table 2. The highest *ZT* (0.018) is reached for Ca_0.98_Gd_0.02_MnO_3−*δ*_, which represents a 125% increase when compared to the CaMnO_3−*δ*_. Lan et al. [30] reported that the *ZT* value is ~0.02 at 300 K for Ca_0.94_Gd_0.06_MnO_3_ prepared by the coprecipitation method and *ZT* value is ~0.018 at 300 K for Ca_0.96_Gd_0.04_MnO_3_ prepared by solid state reaction, which is in agreement with our Ca_0.98_Gd_0.02_MnO_3−*δ*_ with *ZT* = 0.018 at 300 K. These results suggesting that there is scope for improvement of n-type materials for high-temperature thermoelectric application.

## 4. Conclusions 

The thermoelectric properties (*ρ*, *S*, and *κ*) of Ca_1−*x*_Gd_*x*_MnO_3−*δ*_ (*x* = 0.00, 0.02, and 0.05) polycrystalline samples are investigated carefully. All the samples are a single phase with an orthorhombic structure. The iodometric titration results indicate that the electrical resistivity of Ca_1−*x*_Gd_*x*_MnO_3−*δ*_ correlates well with the average valence of the Mn^*v*+^ and oxygen deficiency. The smallest average valence of Mn^*v*+^ for Ca_0.95_Gd_0.05_MnO_3−*δ*_ has the smallest resistivity among the doped samples. The negative thermopower confirms that the domain carriers are electrons for all the samples. Doping of gadolinium on the calcium sites of CaMnO_3−*δ*_ produces reduction of resistivity and thermal conductivity. As a result, Ca_0.98_Gd_0.02_MnO_3−*δ*_ has the highest *ZT* among the doped samples. These results suggest that improving the thermoelectric properties is achived by doping concentration.

## Figures and Tables

**Table 1:** Lattice parameters of the Ca_1−*x*_Gd_*x*_MnO_3−*δ*_ (0.00, 0.02, and 0.05).

Samples	*a* (Å)	*b* (Å)	*c* (Å)
CaMnO_3−*δ*_	5.27 (9)	7.43 (*2*)	5.26 (*1*)
Ca_0.98_Gd_0.02_MnO_3−*δ*_	5.24 (6)	7.44 (*4*)	5.28 (*6*)
Ca_0.98_Gd_0.05_MnO_3−*δ*_	5.26 (*3*)	7.43 (*2*)	5.28 (*3*)

**Table 2:** Room temperature characterization and properties of the Ca_1−*x*_Gd_*x*_MnO_3−*δ*_ (0.00, 0.02, and 0.05).

Samples	Mn^*v+*^	δ	*ρ*	*S*	*κ*_total_	*κ*_el_	*κ*_ph_	PF	ZT
			(mΩ-cm)	(*μ*V/K)	(W/mK)	(W/mK)	(W/mK)	(*μ*W/cm K^2^)	
CaMnO_3−*δ*_	3.90 (8)	−0.04 (*4*)	102	−319	3.72	0.007	3.713	0.99	0.008
Ca_0.98_Gd_0.02_MnO_3−*δ*_	3.92 (8)	−0.02 (*4*)	041	−223	1.99	0.041	1.949	1.21	0.018
Ca_0.98_Gd_0.05_MnO_3−*δ*_	3.88 (9)	−0.03 (*4*)	019	−113	1.26	0.019	1.241	0.67	0.016
